# Bacteraemia during Transurethral Resection of the Prostate: What Are the Risk Factors and Is It More Common than We Think?

**DOI:** 10.1371/journal.pone.0157864

**Published:** 2016-07-08

**Authors:** Amar Raj Mohee, Deborah Gascoyne-Binzi, Robert West, Selina Bhattarai, Ian Eardley, Jonathan A. T. Sandoe

**Affiliations:** 1 Department of Urology, The Christie NHS Foundation Trust, 550 Wilmslow Road, Manchester, United Kingdom; 2 Department of Microbiology, The Leeds Teaching Hospitals NHS Trust, Beckett Street, Leeds, United Kingdom; 3 Department of Biostatistics, The University of Leeds, Woodhouse Lane, Leeds, United Kingdom; 4 Department of Pathology, The Leeds Teaching Hospitals NHS Trust, Beckett Street, Leeds, United Kingdom; 5 Department of Urology, The Leeds Teaching Hospitals NHS Trust, Beckett Street, Leeds, United Kingdom; 6 University of Leeds and Leeds Teaching Hospitals NHS Trust, Beckett Street, Leeds, United Kingdom; King's College London, UNITED KINGDOM

## Abstract

The aim of this work was to investigate the microbial causes, incidence, duration, risk factors and clinical implications of bacteraemia occurring during transurethral resection of the prostate (TURP) surgery to better inform prophylaxis strategies. An ethically approved, prospective, cohort study of patients undergoing TURP was conducted. Clinical information and follow-up details were collected using standardized data collection sheets. Blood was obtained for culture at 6 different time points peri-procedure. Standard of care antibiotic prophylaxis was given prior to surgery. Bacteriuria was assessed in a pre-procedure urine sample. Histopathology from all prostate chips was assessed for inflammation and malignancy. 73 patients were consented and 276 blood samples obtained. No patients developed symptomatic bacteraemia during the procedure, 17 patients developed asymptomatic bacteraemia (23.2%). *Enterococcus faecalis* and *Pseudomonas aeruginosa* were the most common organisms cultured. 10 minutes after the start of the TURP, the odds ratio (OR) of developing bacteraemia was 5.38 (CI 0.97–29.87 p = 0.05), and 20 minutes after the start of the procedure, the OR was 6.46 (CI 1.12–37.24, p = 0.03), compared to before the procedure. We also found an association between the development of intra-operative bacteraemia and recent antibiotic use (OR 4.34, CI 1.14–16.62, p = 0.032), the presence of a urinary catheter (OR 4.92, CI 1.13–21.51, p = 0.034) and a malignant histology (OR 4.90, CI 1.30–18.46, p = 0.019). There was no statistical relationship between pre-operative urine culture results and blood culture results. This study shows that asymptomatic bacteraemia is commonly caused by TURP and occurs in spite of antibiotic prophylaxis. Our findings challenge the commonly held view that urine is the primary source of bacteraemia in TURP-associated sepsis and raise the possibility of occult prostatic infection as a cause of bacteraemia. More work will be needed to determine the significance of transient bacteraemia in relation to more serious complications like infective endocarditis and malignancy.

## Introduction

Transurethral resection of the prostate (TURP) is a common operation (150, 000 per year in US) and is associated with a significant infective morbidity. Reported rates of bacteraemia following TURP without antimicrobial prophylaxis vary widely (0–31%), but decrease to 1% with antimicrobial prophylaxis[1,2]. Anecdotes of severe sepsis following TURP are common[3] and we have recently shown an association between urological procedures and an increased risk of enterococcal endocarditis[4]. A better understanding these infective complications is necessary to design effective prevention and treatment strategies.

Few studies have been specifically designed to assess the frequency and implications of bacteraemia related to TURP; most studies report bacteriuria, urinary tract infections (UTIs) and fever. Only if patients were clinically suspected to have a bacteraemia (e.g. fever or rigors), were blood cultures then taken to determine the presence of bacteraemia. The rate of asymptomatic bacteraemia in TURP patients is not known; only a few old studies, with design limitations, have addressed the issue [5–8].

All major urology guidelines recommend the use of antimicrobial prophylaxis during a TURP[9–11]. However, antimicrobial prophylaxis is used to decrease the incidence of post-operative bacteriuria, rather than other significant infective parameters such as bacteraemia, bloodstream infection or severe sepsis[12–14]. Urological instrumentation is associated with enterococcal endocarditis, (~1 in 4200 procedures), [4] however, it is still not known why some patients develop such severe infective episodes whilst others have an uneventful procedure. It is not known if prostate pathology affects the risk of serious infective complications.

The primary aim of the study was to investigate the incidence, timing, duration and identity of bacteraemia or bloodstream infection occurring during TURP. A secondary aim was to investigate the patient (e.g. underlying prostatic disease) and procedure-related risk factors associated with the development of peri-operative bacteraemia during TURP.

## Materials and Methods

### Ethics

Ethics approval was obtained from the Leeds West research ethics committee (REC number: 10/H1307/5) and institutional approval was also obtained(R&D number: UR09/9173). All patient gave written consent to participate in the study.

### Study design, inclusion and exclusion criteria

For the primary aim, a prospective, cohort study design was undertaken on patients having TURP at our institution. For the secondary aim, a case control design was used where ‘cases’ were those patients who had evidence of bacteraemia and the controls were patients who did not develop bacteraemia. The study included both catheterised patients undergoing TURP for acute urinary retention and non-catheterised patients undergoing TURP for lower urinary tract symptoms (LUTS). Patients with both benign prostatic hyperplasia and known prostatic adenocarcinoma of the prostate were included in the study.

### Definitions

Bacteraemia was defined as the presence of bacteria in blood, irrespective of the bacterial count[15]. Bloodstream infection was defined as bacteraemia with clinical signs or symptoms of infection. Single blood culture isolates of normal skin flora (coagulase-negative staphylococci, coryneforms) were considered likely contaminants. Bacteriuria was defined as >10^4^ cfu/ml microorganisms in pure culture.

### Study size

Previous articles suggest that the incidence during prostatic surgery is 29–46%, we intended to undertake a logistic regression analysis in which case a sample size of at least 69 patients would provide 20 cases—permitting robust analysis for two risk factors.

### Setting and surgical considerations

The Leeds Teaching Hospitals NHS Trust is a tertiary referral centre and teaching hospital of around 2500 beds. It undertakes approximately 250 TURP procedures yearly by 10 urological surgeons. TURP was carried out under general or spinal anaesthesia. Both monopolar and bipolar devices were used for the procedure, dependent on the surgeon’s preference. The local policy for antimicrobial prophylaxis for TURP at our institution was intravenous gentamicin 160mg, within the hour prior to the procedure.

### Demographics and clinical variables

A detailed medical history was obtained pre-procedure, using a structured form, to obtain details of possible risk factors for the development of bacteraemia. The variables collected included age, weight, recent antimicrobial therapy, immunosuppression, diabetes, smoking, co-existing infection, recent long hospital stay, recurrent UTIs, urinary catheter and urolithiasis. A structured telephone interview was conducted with patients three months after the procedure to gather data relating to infective complications. The hospital patient pathway management (PPM) database and the results servers were accessed to confirm the data obtained at the telephone interview.

### Microbiological sampling and processing

#### Blood sampling

**Fig 1:**
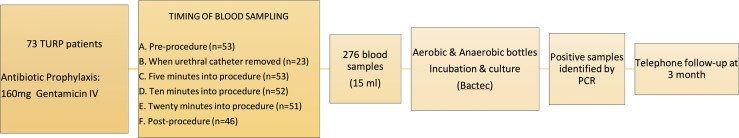
Schematic representation of data acquired from recruited participants.

Blood was collected at 5–6 time-points peri-procedure as shown in Fig 1, with variation depending on urinary catheterisation status pre-procedure. 20ml of blood was obtained at each time point. 15ml of the blood was transferred into Biomerieux BacT/ALERT® aerobic and anaerobic bottles **(**bioMérieux UK Limited, Basingstoke, UK) and all samples were subcultured after 10 days of incubation at 37°C. Any growth detected on sub-culture was identified using 16S PCR (forward primer: AGAGTTTGATCCTGGCTCAG, reverse primer CTACGCATTTCACCGCTACAC) as described previously[16].

#### Urine sampling

Urine samples were plated on blood and CLED agar and incubated at 37.4°C at atmospheric pressure for 24 hours. Bacterial growth detected on agar plates was identified by 16S PCR[16].

### Histopathology

An experienced histopathologist independently reviewed prostate tissue obtained at the TURP to determine the presence of inflammation and calcification, in addition to the presence of malignancy, according to criteria set in the report from the International Society of Urologic Pathology consensus conference[17].

### Statistical analysis

Non-normally distributed, continuous variables are presented as median and range and normally distributed variables, as mean and standard deviations (SD). Pearson’s Chi-Square test was used to evaluate the association between bacteriuria and bacteraemia (SPSS Version 20.0.0, IBM Corporation). A multi-level statistical model was designed, using the open source statistical computing environment, specifically Microsoft R Open 3.2.3 (http://www.r-project.org) to evaluate whether there was an association between the timing of blood collection and the development of bacteraemia (S1 Statistical Model). A complete data set was used for the multiple regression analysis and the analysis was considered exploratory in nature.

Univariable and multivariable analyses were performed to assess the association between the possible risk factors for developing bacteraemia and the development of bacteraemia (SPSS Version 20.0.0, IBM Corporation). A multiple comparison method was not used as the analysis was considered exploratory.

## Results

### Participants

**Fig 2:**
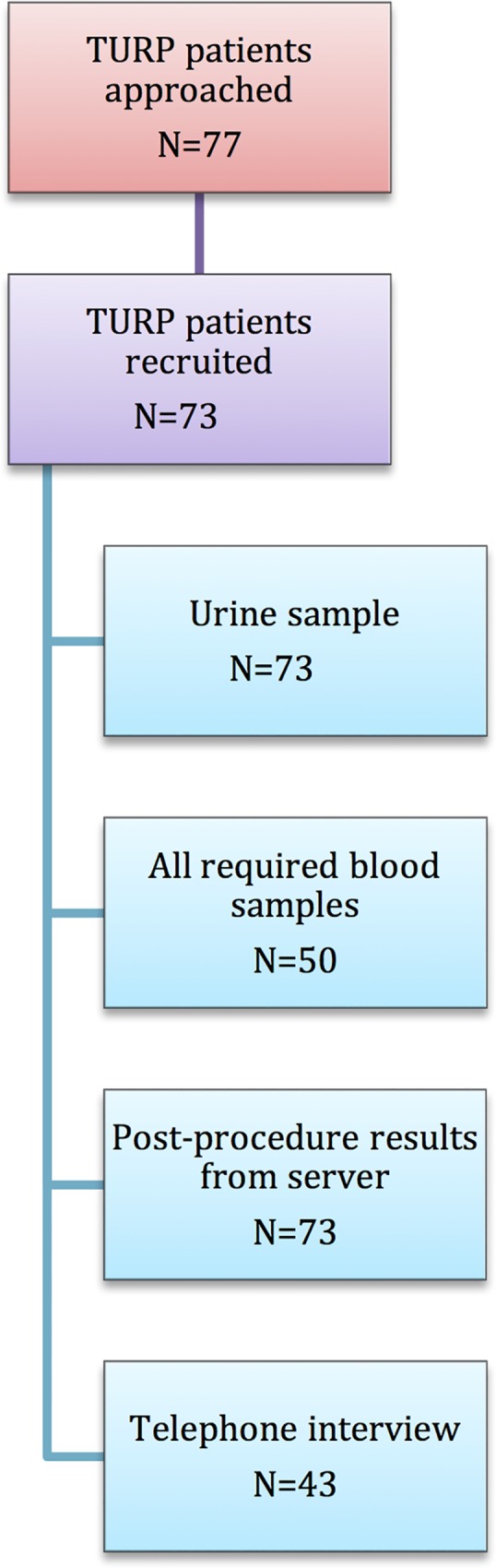
Participants recruited in the prospective study.

77 patients were screened and 74 of these were consented to participate. One patient was found to have a bladder tumour intraoperatively and did not proceed with a TURP operation. Fig 2 shows the number of patients available for each step of the analysis. Table 1 shows the demographics of the 73 patients recruited to the study. Blood samples was obtained from 54 of the 73 patients. A urine sample and prostate histology were available for all 73 patients recruited.

**Table 1:** Demographics of recruited participants having a TURP.

Variable		N = 73
Mean age (years)		72.7±8.56
Mean weight (kg)		86.6±15.3
Within 2 weeks prior to TURP	Antimicrobial use (%)	18 (24.7)
	Urological instrumentation (%)	3 (4.1)
	UTI (%)	10 (13.7)
Immunosuppression (%)		2 (2.7)
Diabetes (%)		6 (8.2)
Smoking (%)		8 (11.0)
Co-existing infection (%)		3 (4.1)
Recent extended hospital stay (%)		20 (27.4)
Recurrent UTIs (%)		9 (12.3)
Urolithiasis (%)		4 (5.5)
Urinary catheter (%)		38 (52.1)
Cardiac history	Arrhythmia (%)	14 (19.2)
	Ischaemic heart disease (%)	13 (17.8)
	Valvular Disease	2 (2.7)
Infective endocarditis (%)		0 (0)
Prosthetic device	Orthopaedic (%)	12 (16.4)
	Cardiovascular (%)	8 (11.0)
	Other (%)	1 (1.4)
Organ transplant (%)		0 (0)

### Patient characteristics

Within 2 weeks prior to the TURP, a quarter of patients had received antimicrobial therapy (n = 18, 24.7%), a UTI (n = 10, 13.7%) or urological instrumentation (n = 3, 4.1%) as shown in Table 1. Twenty patients (27.3%) had experienced a long inpatient hospital stay within the three months prior to the procedure for various medical conditions including urological pathologies. Nine patients (12.3%) had a history of microbiologically confirmed recurrent UTIs prior to the surgery. Thirty-eight patients (52.1%) had a urinary catheter *in situ* at the time of their surgery, with a median duration of the catheterisation being 49 days (range 2 days-12 months). Five patients were performing clean intermittent self catheterization (CISC) Twenty-nine patients (39.7%) had an underlying cardiac pathology with 21 patients (28.8%) having a prosthetic device (coronary stents, prosthetic heart valves, pacemakers, joint prostheses) *in situ*.

### Incidence, timing, frequency and duration of bacteraemia

No patients developed symptomatic bloodstream infection during the TURP but seventeen patients (23.3%) were bacteraemic. Table 2 shows the bacterial isolates. *Enterococcus faecalis* and *Pseudomonas aeruginosa* were the most common organisms cultured. Three patients (4.1%) were bacteremic prior to TURP and ten patients (13.7%) suffered a repeated bacteraemia (the recovery of the same organisms on more than one occasion) during the course of the procedure. Two samples were regarded as contaminants as the organisms detected (*Staphylococcus caprae* and *Corynebacterium glucuronolyticum* respectively) are known skin organisms and were recovered from single blood samples[18].

**Table 2:** Bacteraemia detected in participants having TURP.

Patient	Presence of catheter	Preoperative urine isolate	Identity of bacteraemia	Number of positive time points (n = 39)	Comment
1	No	No isolate	*Staphylococcus caprae*	1	Considered contaminant
2	Yes	*Corynebacterium amycolatum*	*Actimonyces turicensis*	3	
2	Yes	*Enterococcus faecalis + Pseudomonas aeruginosa*	*Pseudomonas aeruginosa*	2	
4	Yes	No isolate	*Staphylococcus epidermidis*	1	Considered a contaminant
5	No (CISC)	*Enterococcus faecalis*	*Staphylococcus epidermidis*	2	
6	No (CISC)	*Klebsiella oxytoca*	*Actinomyces neuii*	5	
7	No	No isolate	*Actinobaculum massiliense*	2	
8	No	No isolate	*Corynebacterium glucuronolyticum*	1	Considered a contaminant
9	Yes	*Pseudomonas aeruginosa*	*Veillonella dispar*	1	
10	Yes	*Proteus mirabilis*	*Proteus vulgaris*	5	
11	Yes	*Enterococcus faecalis*	*Enterococcus faecalis*	4	
12	Yes	No isolate	*Streptococcus anginosus*	1	
13	Yes	*Pseudomonas aeruginosa*	*Pseudomonas aeruginosa*	3	
14	Yes	*Pseudomonas aeruginosa*	*Pseudomonas aeruginosa*	4	
15	Yes	*Enterococcus faecalis*	*Enterococcus faecalis*	1	
16	Yes	No isolate	*Staphylococcus epidermidis*	1	Considered a contaminant
17	Yes	*Pseudomonas aeruginosa*	*Streptococcus agalactiae*	2	

Statistical modeling showed that bacteraemia was most likely to occur 10 and 20 minutes into the procedure; at 10 and 20 minutes, bacteraemia was 5.38 and 6.46 times more likely to be detected respectively, compared to before the start of the procedure (Table 3, S2 Statistical Model). Although fourteen of the seventeen patients (82.4%) who developed bacteraemia during their procedure had a urinary catheter in-situ or were performing CISC prior to the procedure, removal of a urethral catheter prior to instrumentation did not appear to increase the likelihood of developing bacteraemia (OR: 0.76, CI 0.193–3.01, p = 0.69). A patient who had antimicrobial therapy in the two weeks prior to a TURP was 4.3 times less likely to develop bacteraemia than a patient who did not receive antibiotics (OR: 4.34, CI 1.14–16.62 p = 0.032). Having a urinary catheter in situ was associated with the development of intra-TURP bacteraemia (OR: 4.92, CI 1.13–21.51, p = 0.034).

**Table 3:** Results from linear model to evaluate the association between the timing of the blood sampling and the development of bacteraemia, using pre-procedure rate as the comparator.

Variable	Odds ratio	95% Confidence interval	p-value
Pre-procedure (A)	1	-	-
Urethral catheter removal (B)	0.72	0.08–6.73	0.77
5 minutes into procedure (C)	4.04	0.72–22.53	0.11
10 minutes into procedure (D)	5.38	0.97–29.87	0.05
20 minutes into procedure (E)	6.46	1.12–37.24	0.04
Post-procedure (F)	4.16	0.74–23.25	0.10

### Bacteriuria

Thirty-four patients (46.6%) had preoperative bacteriuria, with *Enterococcus faecalis* being the most common organism cultured (Table 4). Eleven of the thirty-four patients (32.3%) with bacteriuria did not have a urinary catheter *in situ*, although four out of those eleven patients performed clean intermittent self-catheterisation (CISC) on a regular basis. Statistical concordance between blood and urine isolates was poor (χ^2^ = 3.74, p = 0.15). However, 6 out of the 17 patients (35.3%) with evidence of bacteremia had the same organism both in blood and urine.

**Table 4:** The identity of bacteriuria prior to TURP.

Identity of bacteriuria	Number of participants without a urinary catheter	Number of participants with a urinary catheter
*Enterococcus faecalis*	3	7
*Pseudomonas aeruginosa*	0	5
*Escherichia coli*	2	2
Coagulase negative *Staphylococcus*	1	2
*Klebsiella oxytoca*	2	0
*Klebsiella penumoniae*	0	2
*Corynebacterium amycolatum*	0	2
*Morganella morganii*	1	0
*Proteus mirabilis*	0	1
*Staphylococcus aureus*	1	0
Unidentified	0	3
TOTAL (n = 34)	10	24

### Prostatic pathology

Histopathology showed 20 (27.4%) patients had prostate cancer and 26 (35.6%) had evidence of prostatic inflammation (23 with chronic inflammation, two with both acute and chronic inflammation, and one with acute inflammation). Three patients (4.1%) had evidence of prostatic calcification. A patient with a malignant histology was 4.9 times more likely to develop bacteraemia than a patient with benign histology (OR: 4.90, CI: 1.30–18.46, p = 0.019) (Table 5, S3 Statistical Model and S1 Raw Data). There was also a statistical association between the development of intraoperative bacteraemia and prostatic inflammation (χ^2^ = 4.97, p = 0.026), but we were unable to fit prostatic inflammation into the multivariable model owing to the multi-level association between antimicrobial use, prostatic malignancy, age and bacteraemia. The frequency of calcification was too low to detect any statistical association with intraoperative bacteraemia.

**Table 5:** Results from multivariable analysis to assess the association between the development of bacteraemia and risk factors.

	OR	95% C.I. for OR		P-value
		Lower	Upper	
Antibiotics Use	4.34	1.13	0.032	16.62
Urethral catheter in situ	4.92	1.13	0.034	21.51
Malignancy on histology	4.90	1.30	0.019	18.46

### Follow-up

52 patients (71.2%) patients were discharged within two days of the TURP procedure, with haematuria being the main reason for delay in discharge from the hospital as shown in Table 6. Two patients had symptoms/signs of systemic infection post TURP requiring antimicrobial therapy, although there was no intra-procedure bacteraemia in these two patients. The organisms grown in blood cultures, collected during the pyrexial episodes, were *Staphylococcus epidermidis* (considered a likely contaminant) and *Enterococcus faecalis*. The preoperative urine culture for the former grew *Staphylococcus hominis* while no isolate was retrieved from the urine sample of the latter. Forty-one patients (56.1%) had a documented bacteriuria within the three months after the procedure. Forty-three (58.9%) of the recruited patients were contactable by telephone for an interview 3 months post procedure. Sixteen (21.9%) complained of lower urinary tract symptoms post procedure. Thirteen patients (17.8%) were readmitted to the hospital (Table 6).

**Table 6:** Post procedure parameters for participants undergoing TURP.

**Parameters from hospital server**		**n = 73**
Patients discharged within 2/7		52
Patients with positive MSU within 3 months of procedure		41
Patients with a positive blood culture within 3 month of the procedure		7
Malignant prostate histology		20
**Parameters from telephone interview**		**n = 43**
Felt unwell		15
Lower urinary tract symptoms		16
Urine sample to GP		15
Antibiotics from GP		15
Readmission (within 3 months)	For Infection	7
	For other causes	6

## Discussion

Understanding the pathogenesis and implications of bacteraemia during TURP is important to the design of effective preventative measures. We found that bacteraemia was common, affecting 23% of our patients, in spite of antimicrobial prophylaxis. Bacteraemia mostly occurred after the insertion of the instrument used for resection of the prostate and did not usually last for the duration of the procedure. The finding that bacteraemia most often developed between 10 to 20 minutes after the start of the procedure and reduced after the end of the procedure suggests that there is a causal relationship between TURP and bacteraemia. However, TURP did not explain the bacteraemia in all patients because some were bacteraemic prior to the start of the procedure. Ten of the seventeen-bacteraemic patients had persistent bacteraemia during the resection of the prostate.

We found an association between bacteraemia and prior urinary catheterization, prior antimicrobial use and malignant histology, but no conclusions about causation can be made. It is believed that some patient groups, such as those with a urethral catheter *in situ* pre-operatively are at increased risk of developing infective episodes post TURP[19]. Whilst the presence of a urinary catheter pre-operation was a risk factor for bacteraemia, it did not explain all cases and there was a poor correlation between pre-operative urine culture results and the microorganisms found in the blood. For these reasons we speculate that the source of the bacteraemia might be the prostate in some cases. Bacteraemia might occur as a consequence of trauma to the prostate tissue during the resection rather than resulting from the removal of a ‘colonised’ urethral catheter. [20] [21]. While 46.6% of our patients had bacteriuria (with two-thirds of these patients having a urinary catheter in-situ), there was no concordance between urine culture results and bacteraemia. Given that the same isolate was retrieved from the urine and blood of 35.3% of the patients with intra-operative bacteremia, it is possible that some of the bacteraemias resulted from the prior bacteriuria. This finding was confirmed by Okhawa *et al*. who showed that bacteraemia peri-prostatic surgery was more prevalent in patient with pre-operative bacteriuria than patients with sterile urine (53.7% vs 8.2%)[22]. The association with previous antibiotic therapy suggests that these patients may have presented with symptoms or signs of infection before they had surgery.

The bacteraemia was ‘controlled’ in all of the patients in this study; none of the bacteraemic patients developed symptoms or signs of systemic infection. The lack of patients with bloodstream infection or severe sepsis in this cohort probably reflects the small sample size and is consistent with studies of antimicrobial prophylaxis for TURP which reported severe sepsis and bloodstream infection in 1–5% of cases[8,23,24]. Demonstrating the presence of asymptomatic bacteraemia in patients undergoing TURP in spite of antimicrobial prophylaxis raises concerns about the relevance of our current prophylaxis regimen. However, we do not know if the rates of bloodstream infection and severe sepsis would be higher without prophylaxis and the short duration of bacteraemia in some cases may be influenced by administration of prophylaxis. It is surprising that *Pseudomonas aeruginosa* was a frequent cause of bacteraemia in this analysis in spite of use of a prophylactic agent (gentamicin) that would be expected to have anti-pseudomonal activity. Conversely, it is not surprising that gentamicin did not prevent enterococcal bacteremia. The long-term effects of this asymptomatic bacteraemia are not known and we feel that this exploratory study does not provide sufficient evidence to drive a change in prescribing practice. However, we recently published data demonstrating an association between prior urological procedures and the subsequent development of enterococcal infective endocarditis[4] with an estimated rate of enterococcal IE of fewer than 1 endocarditis case every 4000 procedures. Several potential endocarditis-causing bacteria isolates were obtained from blood of the participants in our study. It is possible that inserting an instrument in the prostate has long-term infective consequences, mediated by a silent bacteraemia, but further work would be required to investigate this.

This is the first study to demonstrate an association between prostatic inflammation and malignancy and bacteraemia during TURP and it suggests that bacteria within the prostate might be the source of peri-operative bacteraemia, rather than the traditionally held view that bacteria in urine are responsible. This in turn raises questions about the possible role of chronic low-grade prostatic infection in prostatic malignancy, a possibility that has been raised before[25].

There were unexpected microbiological results including repeated isolation of *Actinomyces* species in two patients. There is only one report of the association of *Actinomyces* and prostatic infection in the literature[26]. We probably recovered these bacteria because of the prolonged blood culture protocol with routine subcultures. We used this protocol in the research setting to maximize the sensitivity of the assay as sample processing was blinded to the clinical team and would not be used to manage patient care. Routine microbiology protocols for blood cultures in the UK (usual incubation is 5–7 days) would not have detected these bacteria. This finding adds weight to the hypothesis that undiagnosed, chronic low-grade prostatic infection might have an insidious role in patients with lower urinary tract symptoms or prostatic malignancy.

Although we have demonstrated asymptomatic bacteraemia during TURP, this is just a ‘snapshot’ of the longitudinal history of the patient. Our results support the role of transurethral prostatic surgery as a cause of bacteraemia, but it may be that patients with prostatic pathology have recurrent asymptomatic bacteraemias, outside the setting of the operating theatre. In the context of serious infective complications of TURP, such as IE, the cumulative effect of recurrent spontaneous bacteraemias might be more important in pathogenesis than the brief episode in relation to TURP, as has been postulated in the pathogenesis of dental-related IE[27]. It may be possible that the prostate gland is not a bacteria-free organ in the setting of urological pathology. Although, there are a few studies looking at culture of prostate chips and biopsies, newer methodologies like PCR and electron microscopy may help shed light on whether bacteria resides in or on the prostate and whether they reside as colonies or biofilms that are hard to culture[21].

Though this is the largest series to date to assess bacteraemia in TURP patients, the results may not be applicable to the general population undergoing TURP because the sample size is small. We recruited to target, but found the incidence of bacteraemia to be lower than expected, reducing the power to investigate risk factors. The authors acknowledge it would have been useful to evaluate other variables (e.g prostate chip culture), but this only became apparent at the end of the study. The wide confidence intervals reflect the small sample size and highlight the need for further work with larger numbers of patients.

The authors also acknowledge that only 54 patients of the 73 patients recruited in the study provided blood samples. This discrepancy may be explained by a number of reasons. Firstly, some patients did not proceed to surgery owing to unavailability of a hospital bed or the anaesthetist deeming the patient not fit for surgery. Secondly, for the patients who proceeded to surgery, the principal investigator was unable to gain adequate venous access to obtain the required blood samples.

## Conclusion

We have demonstrated a high rate of bacteraemia in patients undergoing TURP that was associated to the prostatic surgery rather than urethral catheter manipulations. Bacteraemia occurred in spite of antimicrobial prophylaxis. A range of human pathogens was identified including several known to cause chronic infections, prostatitis and infective endocarditis. The association between peri-TURP bacteraemia and prostatic adenocarcinoma and inflammation raises the possibility of bacterial involvement in the pathogenesis of both bladder outflow obstruction and prostate adenocarcinoma. This warrants further investigation.

## Supporting Information

S1 Raw Data Data used to construct statistical model in S3 Statistical Model. (CSV)

S1 Statistical Model Description of the construction of the statistical model. (DOCX)

S2 Statistical Model Statistical analysis of the prevalence of bacteraemia during TURP. (PDF)

S3 Statistical Model Construction of linear model to evaluate risk factors associated with the development of bacteraemia. (PDF)
