# Supplementary material for: Bacteraemia during Transurethral Resection of the Prostate: What Are the Risk Factors and Is It More Common than We Think?
Source: PLoS One. 2016 Jul 8;11(7):e0157864. doi: 10.1371/journal.pone.0157864 (PMC4938130; doi:10.1371/journal.pone.0157864)
Supplement: S1 Statistical Model — (DOCX) [file pone.0157864.s002.docx]

Supporting Evidence S4

A multi-level statistical model was designed , using the open source statistical computing environment, specifically Microsoft R Open 3.2.3 (<http://www.r-project.org>) to evaluate whether there was an association between the timing of blood collection and the development of bacteraemia. A complete data set was used for the multiple regression analysis. Both hypothesised and evidenced variables were included as covariates in a logistic regression for bacteremia. Model building started with consideration of categorised variables.  All subsets of these variables were fitted and the best fit, defined by lowest AIC, was taken forward.  Library bestglm was employed for analysis. Continuous covariates, age and weight were next added to this model using splines so that nonlinear behaviour was considered, using library mgcv.  As an increase in AIC was observed, these covariates were then dropped. Further categorisation of urinary catheter was considered by including the duration of use.  As an increase in AIC was seen, the model reverted to include only the presence/absence of urinary catheter. Due to the limited size of the dataset (73 observations with 17 occurrences of bacteraemia), only the main effects were considered; no interaction terms were modelled.

Limitations

The model selection procedure based on all subsets, viable since there were only a small number of factors to be considered, may have resulted in narrower confidence intervals and coefficients that would require some shrinkage. However, given the size of the effects and the level of significance of the selected factors, the overall conclusions were most unlikely to change.
