# Supplementary material for: Bacteraemia during Transurethral Resection of the Prostate: What Are the Risk Factors and Is It More Common than We Think?
Source: PLoS One. 2016 Jul 8;11(7):e0157864. doi: 10.1371/journal.pone.0157864 (PMC4938130; doi:10.1371/journal.pone.0157864)
Supplement: S2 Statistical Model — (PDF) [file pone.0157864.s003.pdf]

Prevalence of bacteremia during TURP

Robert M West

Thursday, February 18, 2016

```r
setwd("F:\\TURP") # Select the working directory
turp.patients <- read.csv("BacteremiaTURP.csv")
library(lme4)  # So that random effects can be fitted
```

```
## Loading required package: Matrix
```

```r
number.patients <- length(unique(turp.patients$sample)) # Check the correct number of patients - 54
```

```r
model <- glmer(Bacteremia~time.pt+(1|sample), turp.patients, family=binomial())
summary(model)
```

```
## Generalized linear mixed model fit by maximum likelihood (Laplace
##   Approximation) [glmerMod]
##  Family: binomial  ( logit )
## Formula: Bacteremia ~ time.pt + (1 | sample)
##    Data: turp.patients
##
##      AIC      BIC   logLik deviance df.resid
##    189.5    214.7    -87.7    175.5      263
##
## Scaled residuals:
##     Min      1Q  Median      3Q     Max
## -1.8924 -0.1236 -0.0992 -0.0486  4.7787
##
## Random effects:
##  Groups Name        Variance Std.Dev.
##  sample (Intercept) 11.19    3.345
## Number of obs: 270, groups:  sample, 53
##
## Fixed effects:
##             Estimate Std. Error z value Pr(>|z|)
## (Intercept)  -5.4975     1.6416  -3.349 0.000811 ***
## time.ptB     -0.3259     1.1388  -0.286 0.774725
## time.ptC      1.3961     0.8769   1.592 0.111360
## time.ptD      1.6823     0.8749   1.923 0.054483 .
## time.ptE      1.8661     0.8935   2.088 0.036758 *
## time.ptF      1.4259     0.8777   1.625 0.104261
## ---
## Signif. codes:  0 '***' 0.001 '**' 0.01 '*' 0.05 '.' 0.1 ' ' 1
##
## Correlation of Fixed Effects:
##          (Intr) tm.ptB tm.ptC tm.ptD tm.ptE
## time.ptB -0.195
## time.ptC -0.479  0.436
## time.ptD -0.510  0.434  0.652
## time.ptE -0.521  0.425  0.644  0.658
## time.ptF -0.468  0.436  0.639  0.649  0.640
```

```r
coef.table <- as.data.frame(coef(summary(model)))
coef.table$OR <- exp(coef.table$Estimate)
coef.table$Lower <- with(coef.table, exp(Estimate - `Std. Error` * sqrt(qchisq(0.95, 1))))
coef.table$Upper <- with(coef.table, exp(Estimate + `Std. Error` * sqrt(qchisq(0.95, 1))))
coef.table[ ,c(5,6,7,4)] # To report results
```

```
##                      OR        Lower      Upper     Pr(>|z|)
## (Intercept) 0.004096829 0.0001641161  0.1022691 0.0008111014
## time.ptB    0.721868241 0.0774716158  6.7262539 0.7747254928
## time.ptC    4.039376385 0.7242894431 22.5276811 0.1113600265
## time.ptD    5.378016735 0.9681575954 29.8743347 0.0544832587
## time.ptE    6.462865028 1.1216511889 37.2385148 0.0367575840
## time.ptF    4.161644696 0.7449684341 23.2483496 0.1042608416
```

```r
var.re <- as.numeric(summary(model)$varcor)
estimated.ICC <- var.re/(var.re+ pi*pi/3)
estimated.ICC #  Check
```

```
## [1] 0.7727591
```
