# Supplementary material for: Bacteraemia during Transurethral Resection of the Prostate: What Are the Risk Factors and Is It More Common than We Think?
Source: PLoS One. 2016 Jul 8;11(7):e0157864. doi: 10.1371/journal.pone.0157864 (PMC4938130; doi:10.1371/journal.pone.0157864)
Supplement: S3 Statistical Model — (PDF) [file pone.0157864.s004.pdf]

TURP analysis

RM West

Monday, February 18, 2016

```
library(epibasix)
library(bestglm)
```

```
## Loading required package: leaps
```

```
library(mgcv)
```

```
## Loading required package: nlme
## This is mgcv 1.8-10. For overview type 'help("mgcv-package")'.
```

```
setwd("F:\\TURP")
turp <- read.csv("TURPdata.csv")
```

```
turp$Over.75 <- ifelse(turp$Age>74.99, "Over 75", "Under 75")
turp$Over.75 <- factor(turp$Over.75, levels=c("Under 75", "Over 75"))
turp$Over.85.kg <- ifelse(turp$Weight>84.99, "Over 85 kg", "Under 85 kg")
turp$Over.75 <- factor(turp$Over.85.kg, levels=c("Under 85 kg", "Over 85 kg"))
turp$Cardiac.Hx <- ifelse(substr(turp$Cardiac.History,1,1)=="N", "No", "Yes")
```

```
tab1 <- with(turp, table(Over.75, Bacteraemia))
tab1
```

```
##               Bacteraemia
## Over.75         0  1
##   Under 85 kg 29  5
##   Over 85 kg  27 10
```

```
epi2x2(tab1)
```

```
## Epidemiological 2x2 Table Analysis
##
## Input Matrix:
##               Bacteraemia
## Over.75         0  1
##   Under 85 kg 29  5
##   Over 85 kg  27 10
##
## Pearson Chi-Squared Statistic (Includes Yates' Continuity Correction): 0.959
## Associated p.value for H0: There is an association between exposure and outcome vs. HA: No associati
## p.value using Fisher's Exact Test: 0.252
##
## Estimate of Odds Ratio: 2.148
## 95% Confidence Limits for true Odds Ratio are: [0.65, 7.094]
```

```r
tab1 <- with(turp, table(Over.85.kg, Bacteraemia))
tab1
```

```
##              Bacteraemia
## Over.85.kg     0  1
##   Over 85 kg  27 10
##   Under 85 kg 29  5
```

```r
epi2x2(tab1)
```

```
## Epidemiological 2x2 Table Analysis
##
## Input Matrix:
##              Bacteraemia
## Over.85.kg     0  1
##   Over 85 kg  27 10
##   Under 85 kg 29  5
##
## Pearson Chi-Squared Statistic (Includes Yates' Continuity Correction): 0.959
## Associated p.value for H0: There is an association between exposure and outcome vs. HA: No associati
## p.value using Fisher's Exact Test: 0.252
##
## Estimate of Odds Ratio: 0.466
## 95% Confidence Limits for true Odds Ratio are: [0.141, 1.537]
```

```r
tab1 <- with(turp, table(Prior.Antibiotics, Bacteraemia))
tab1
```

```
##                  Bacteraemia
## Prior.Antibiotics  0  1
##                 0 47  8
##                 1 10  8
```

```r
epi2x2(tab1)
```

```
## Epidemiological 2x2 Table Analysis
##
## Input Matrix:
##                  Bacteraemia
## Prior.Antibiotics  0  1
##                 0 47  8
##                 1 10  8
##
## Pearson Chi-Squared Statistic (Includes Yates' Continuity Correction): 5.445
## Associated p.value for H0: There is an association between exposure and outcome vs. HA: No associati
## p.value using Fisher's Exact Test: 0.018
##
## Estimate of Odds Ratio: 4.7
## 95% Confidence Limits for true Odds Ratio are: [1.424, 15.515]
```

```r
tab1 <- with(turp, table(Urinary.Catheter, Bacteraemia))
tab1
```

```
##                 Bacteraemia
## Urinary.Catheter  0  1
##                0 32  3
##                1 25 13
```

```r
epi2x2(tab1)
```

```
## Epidemiological 2x2 Table Analysis
##
## Input Matrix:
##                 Bacteraemia
## Urinary.Catheter  0  1
##                0 32  3
##                1 25 13
##
## Pearson Chi-Squared Statistic (Includes Yates' Continuity Correction): 5.58
## Associated p.value for H0: There is an association between exposure and outcome vs. HA: No associati
## p.value using Fisher's Exact Test: 0.011
##
## Estimate of Odds Ratio: 5.547
## 95% Confidence Limits for true Odds Ratio are: [1.424, 21.612]
```

```r
tab1 <- with(turp, table(Instrumentation, Bacteraemia))
tab1
```

```
##                Bacteraemia
## Instrumentation  0  1
##               N 55 15
##               Y  2  1
```

```r
epi2x2(tab1)
```

```
## Epidemiological 2x2 Table Analysis
##
## Input Matrix:
##                Bacteraemia
## Instrumentation  0  1
##               N 55 15
##               Y  2  1
##
## Pearson Chi-Squared Statistic (Includes Yates' Continuity Correction): 0.05
## Associated p.value for H0: There is an association between exposure and outcome vs. HA: No associati
## p.value using Fisher's Exact Test: 0.53
##
## Estimate of Odds Ratio: 1.833
## 95% Confidence Limits for true Odds Ratio are: [0.155, 21.619]
```

```r
tab1 <- with(turp, table(Prior.UTI, Bacteraemia))
tab1
```

```
##          Bacteraemia
## Prior.UTI  0  1
##         N 52 11
##         Y  5  5
```

```r
epi2x2(tab1)
```

```
## Epidemiological 2x2 Table Analysis
##
## Input Matrix:
##          Bacteraemia
## Prior.UTI  0  1
##         N 52 11
##         Y  5  5
##
## Pearson Chi-Squared Statistic (Includes Yates' Continuity Correction): 3.607
## Associated p.value for H0: There is an association between exposure and outcome vs. HA: No associati
## p.value using Fisher's Exact Test: 0.035
##
## Estimate of Odds Ratio: 4.727
## 95% Confidence Limits for true Odds Ratio are: [1.166, 19.168]
```

```r
tab1 <- with(turp, table(Immunosuppressant, Bacteraemia))
tab1
```

```
##                    Bacteraemia
## Immunosuppressant    0  1
##                 N   55 16
##                 Y    2  0
```

```r
epi2x2(tab1)
```

```
## Epidemiological 2x2 Table Analysis
##
## Input Matrix:
##                    Bacteraemia
## Immunosuppressant    0  1
##                 N   55 16
##                 Y    2  0
##
## Pearson Chi-Squared Statistic (Includes Yates' Continuity Correction): 0.011
## Associated p.value for H0: There is an association between exposure and outcome vs. HA: No associati
## p.value using Fisher's Exact Test: 1
##
## Estimate of Odds Ratio: 0
## 95% Confidence Limits for true Odds Ratio are: [0, NaN]
```

```r
tab1 <- with(turp, table(Diabetes, Bacteraemia))
tab1
```

```
##           Bacteraemia
## Diabetes    0  1
##   N        52 15
##   Y         5  1
```

```r
epi2x2(tab1)
```

```
## Epidemiological 2x2 Table Analysis
##
## Input Matrix:
##           Bacteraemia
## Diabetes    0  1
##   N        52 15
##   Y         5  1
##
## Pearson Chi-Squared Statistic (Includes Yates' Continuity Correction): 0.036
## Associated p.value for H0: There is an association between exposure and outcome vs. HA: No associati
## p.value using Fisher's Exact Test: 1
##
## Estimate of Odds Ratio: 0.693
## 95% Confidence Limits for true Odds Ratio are: [0.075, 6.4]
```

```r
tab1 <- with(turp, table(Smoking, Bacteraemia))
tab1
```

```
##          Bacteraemia
## Smoking    0  1
##   N       50 15
##   Y        7  1
```

```r
epi2x2(tab1)
```

```
## Epidemiological 2x2 Table Analysis
##
## Input Matrix:
##          Bacteraemia
## Smoking    0  1
##   N       50 15
##   Y        7  1
##
## Pearson Chi-Squared Statistic (Includes Yates' Continuity Correction): 0.053
## Associated p.value for H0: There is an association between exposure and outcome vs. HA: No associati
## p.value using Fisher's Exact Test: 0.676
##
## Estimate of Odds Ratio: 0.476
## 95% Confidence Limits for true Odds Ratio are: [0.054, 4.184]
```

```r
tab1 <- with(turp, table(Coexisting.Infection, Bacteraemia))
tab1
```

```
##                         Bacteraemia
## Coexisting.Infection     0  1
##                    N    54 16
##                    Y     3  0
```

```r
epi2x2(tab1)
```

```
## Epidemiological 2x2 Table Analysis
##
## Input Matrix:
##                         Bacteraemia
## Coexisting.Infection     0  1
##                    N    54 16
##                    Y     3  0
##
## Pearson Chi-Squared Statistic (Includes Yates' Continuity Correction): 0.05
## Associated p.value for H0: There is an association between exposure and outcome vs. HA: No associati
## p.value using Fisher's Exact Test: 1
##
## Estimate of Odds Ratio: 0
## 95% Confidence Limits for true Odds Ratio are: [0, NaN]
```

```r
tab1 <- with(turp, table(Recent.Hospital, Bacteraemia))
tab1
```

```
##                Bacteraemia
## Recent.Hospital  0  1
##               0 44  9
##               1 13  7
```

```r
epi2x2(tab1)
```

```
## Epidemiological 2x2 Table Analysis
##
## Input Matrix:
##                Bacteraemia
## Recent.Hospital  0  1
##               0 44  9
##               1 13  7
##
## Pearson Chi-Squared Statistic (Includes Yates' Continuity Correction): 1.803
## Associated p.value for H0: There is an association between exposure and outcome vs. HA: No associati
## p.value using Fisher's Exact Test: 0.119
##
## Estimate of Odds Ratio: 2.632
## 95% Confidence Limits for true Odds Ratio are: [0.821, 8.444]
```

```r
tab1 <- with(turp, table(Recurrent.UTIs, Bacteraemia))
tab1
```

```
##                 Bacteraemia
## Recurrent.UTIs   0  1
##   N             50 14
##   Y              7  2
```

```r
epi2x2(tab1)
```

```
## Epidemiological 2x2 Table Analysis
##
## Input Matrix:
##                 Bacteraemia
## Recurrent.UTIs   0  1
##   N             50 14
##   Y              7  2
##
## Pearson Chi-Squared Statistic (Includes Yates' Continuity Correction): 0.165
## Associated p.value for H0: There is an association between exposure and outcome vs. HA: No associati
## p.value using Fisher's Exact Test: 1
##
## Estimate of Odds Ratio: 1.02
## 95% Confidence Limits for true Odds Ratio are: [0.19, 5.472]
```

```r
tab1 <- with(turp, table(Urolithiasis, Bacteraemia))
tab1
```

```
##                 Bacteraemia
## Urolithiasis     0  1
##   N             53 16
##   Y              4  0
```

```r
epi2x2(tab1)
```

```
## Epidemiological 2x2 Table Analysis
##
## Input Matrix:
##                 Bacteraemia
## Urolithiasis     0  1
##   N             53 16
##   Y              4  0
##
## Pearson Chi-Squared Statistic (Includes Yates' Continuity Correction): 0.219
## Associated p.value for H0: There is an association between exposure and outcome vs. HA: No associati
## p.value using Fisher's Exact Test: 0.57
##
## Estimate of Odds Ratio: 0
## 95% Confidence Limits for true Odds Ratio are: [0, NaN]
```

```r
tab1 <- with(turp, table(Urinary.Catheter, Bacteraemia))
tab1
```

```
##                  Bacteraemia
## Urinary.Catheter  0  1
##                 0 32  3
##                 1 25 13
```

```r
epi2x2(tab1)
```

```
## Epidemiological 2x2 Table Analysis
##
## Input Matrix:
##                  Bacteraemia
## Urinary.Catheter  0  1
##                 0 32  3
##                 1 25 13
##
## Pearson Chi-Squared Statistic (Includes Yates' Continuity Correction): 5.58
## Associated p.value for H0: There is an association between exposure and outcome vs. HA: No associati
## p.value using Fisher's Exact Test: 0.011
##
## Estimate of Odds Ratio: 5.547
## 95% Confidence Limits for true Odds Ratio are: [1.424, 21.612]
```

```r
tab1 <- with(turp, table(Catheter.Duration, Bacteraemia))
tab1
```

```
##                  Bacteraemia
## Catheter.Duration  0  1
##                   28  2
##          CISC      4  1
##          LT 1m    11  5
##          Over 1m 14  8
```

```r
epi2x2(tab1[c(1,2),])
```

```
## Epidemiological 2x2 Table Analysis
##
## Input Matrix:
##                  Bacteraemia
## Catheter.Duration  0 1
##                   28 2
##             CISC   4 1
##
## Pearson Chi-Squared Statistic (Includes Yates' Continuity Correction): 0.015
## Associated p.value for H0: There is an association between exposure and outcome vs. HA: No associati
## p.value using Fisher's Exact Test: 0.38
##
## Estimate of Odds Ratio: 3.5
## 95% Confidence Limits for true Odds Ratio are: [0.255, 48.032]
```

```r
epi2x2(tab1[c(1,3),])
```

```
## Epidemiological 2x2 Table Analysis
##
## Input Matrix:
##                   Bacteraemia
## Catheter.Duration  0 1
##                    28 2
##              LT 1m 11 5
##
## Pearson Chi-Squared Statistic (Includes Yates' Continuity Correction): 3.168
## Associated p.value for H0: There is an association between exposure and outcome vs. HA: No associati
## p.value using Fisher's Exact Test: 0.04
##
## Estimate of Odds Ratio: 6.364
## 95% Confidence Limits for true Odds Ratio are: [1.071, 37.81]
```

```r
epi2x2(tab1[c(1,4),])
```

```
## Epidemiological 2x2 Table Analysis
##
## Input Matrix:
##                   Bacteraemia
## Catheter.Duration  0 1
##                    28 2
##            Over 1m 14 8
##
## Pearson Chi-Squared Statistic (Includes Yates' Continuity Correction): 5.421
## Associated p.value for H0: There is an association between exposure and outcome vs. HA: No associati
## p.value using Fisher's Exact Test: 0.012
##
## Estimate of Odds Ratio: 8
## 95% Confidence Limits for true Odds Ratio are: [1.495, 42.798]
```

```r
tab1 <- with(turp, table(Cardiac.Hx, Bacteraemia))
tab1
```

```
##           Bacteraemia
## Cardiac.Hx  0  1
##        No  35  9
##        Yes 22  7
```

```r
epi2x2(tab1)
```

```
## Epidemiological 2x2 Table Analysis
##
## Input Matrix:
##           Bacteraemia
## Cardiac.Hx  0  1
##        No  35  9
##        Yes 22  7
```

```
##
## Pearson Chi-Squared Statistic (Includes Yates' Continuity Correction): 0.007
## Associated p.value for H0: There is an association between exposure and outcome vs. HA: No associati
## p.value using Fisher's Exact Test: 0.776
##
## Estimate of Odds Ratio: 1.237
## 95% Confidence Limits for true Odds Ratio are: [0.403, 3.802]
```

```
turp.data <- data.frame(with(turp, cbind(Prior.Antibiotics, Urinary.Catheter,  Prior.UTI, Diabetes,  Hi
turp.data <- na.omit(turp.data) # Check that there is no missing data for model selection procedure
best.AIC.model <- bestglm(turp.data, IC="AIC", family=binomial())
```

```
## Morgan-Tatar search since family is non-gaussian.
```

```
best.AIC.model
```

```
## AIC
## BICq equivalent for q in (0.446891293449154, 0.837384366314189)
## Best Model:
##                       Estimate Std. Error   z value     Pr(>|z|)
## (Intercept)          -3.418718  0.8122956 -4.208711 2.568313e-05
## Prior.Antibiotics1    1.468581  0.6848091  2.144512 3.199190e-02
## Urinary.Catheter1     1.593923  0.7524548  2.118297 3.414988e-02
## Histology1            1.589763  0.6764072  2.350305 1.875802e-02
```

```
model <- glm(Bacteraemia~Prior.Antibiotics+Urinary.Catheter+Histology, data=turp, family=binomial())
summary(model)
```

```
##
## Call:
## glm(formula = Bacteraemia ~ Prior.Antibiotics + Urinary.Catheter +
##     Histology, family = binomial(), data = turp)
##
## Deviance Residuals:
##     Min       1Q   Median       3Q      Max
## -1.0794  -0.5468  -0.2539  -0.2539   2.6271
##
## Coefficients:
##                     Estimate Std. Error z value Pr(>|z|)
## (Intercept)          -3.4187     0.8123  -4.209 2.57e-05 ***
## Prior.Antibiotics     1.4686     0.6848   2.145   0.0320 *
## Urinary.Catheter      1.5939     0.7525   2.118   0.0341 *
## Histology             1.5898     0.6764   2.350   0.0188 *
## ---
## Signif. codes:  0 '***' 0.001 '**' 0.01 '*' 0.05 '.' 0.1 ' ' 1
##
## (Dispersion parameter for binomial family taken to be 1)
##
##     Null deviance: 76.776  on 72  degrees of freedom
## Residual deviance: 59.297  on 69  degrees of freedom
## AIC: 67.297
##
## Number of Fisher Scoring iterations: 5
```

```r
coef.table <- as.data.frame(coef(summary(model)))
coef.table$OR <- exp(coef.table$Estimate)
coef.table$Lower <- with(coef.table, exp(Estimate - `Std. Error` * sqrt(qchisq(0.95, 1))))
coef.table$Upper <- with(coef.table, exp(Estimate + `Std. Error` * sqrt(qchisq(0.95, 1))))
coef.table[ ,c(5,6,7,4)]
```

```
##                           OR      Lower      Upper     Pr(>|z|)
## (Intercept)       0.03275441 0.00666565  0.1609522 2.568313e-05
## Prior.Antibiotics 4.34306891 1.13471330 16.6229192 3.199190e-02
## Urinary.Catheter  4.92302492 1.12652626 21.5140785 3.414988e-02
## Histology         4.90258900 1.30216688 18.4579866 1.875802e-02
```

```r
model <- gam(Bacteraemia~s(Age)+s(Weight)+Prior.Antibiotics+Urinary.Catheter+Histology, data=turp, famil
summary(model)  #  These terms even with nonlinearity add little - higher AIC
```

```
##
## Family: binomial
## Link function: logit
##
## Formula:
## Bacteraemia ~ s(Age) + s(Weight) + Prior.Antibiotics + Urinary.Catheter +
##     Histology
##
## Parametric coefficients:
##                   Estimate Std. Error z value Pr(>|z|)
## (Intercept)        -4.1209     1.0750  -3.833 0.000126 ***
## Prior.Antibiotics   1.3663     0.8125   1.682 0.092635 .
## Urinary.Catheter    2.2547     1.0656   2.116 0.034348 *
## Histology           1.8757     0.8876   2.113 0.034581 *
## ---
## Signif. codes:  0 '***' 0.001 '**' 0.01 '*' 0.05 '.' 0.1 ' ' 1
##
## Approximate significance of smooth terms:
##             edf Ref.df Chi.sq p-value
## s(Age)    1.000   1.00  0.007   0.934
## s(Weight) 6.086   7.23  7.726   0.410
##
## R-sq.(adj) =  0.268   Deviance explained = 36.7%
## UBRE = -0.034768  Scale est. = 1         n = 71
```

```r
plot(model, pages=1)
```

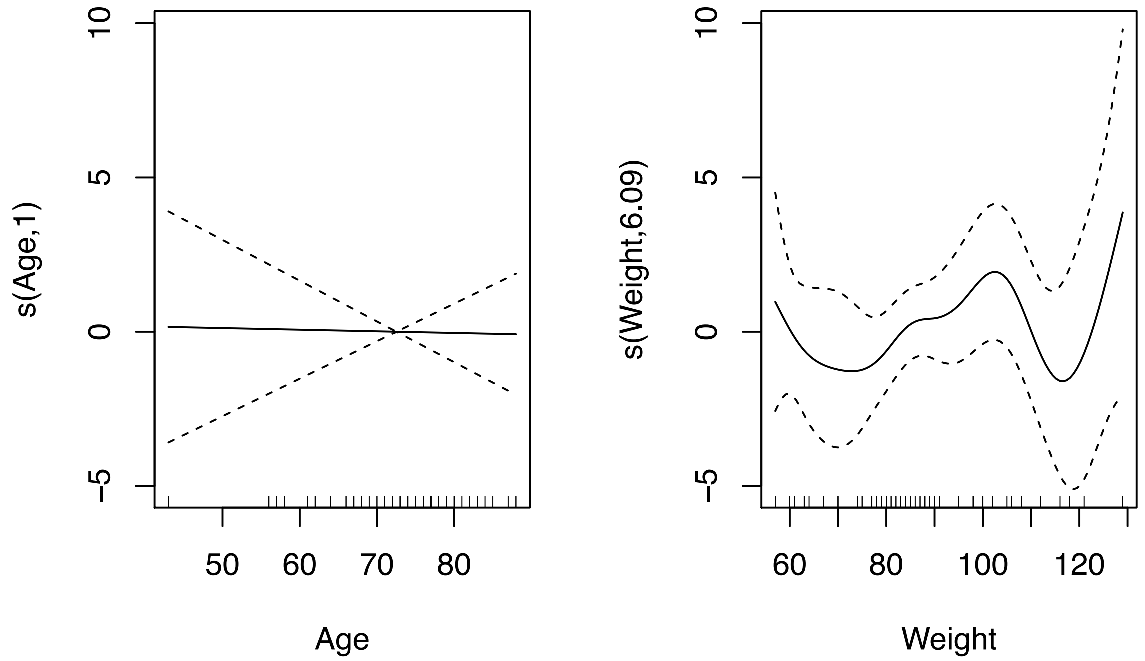


```
model <- glm(Bacteraemia~Prior.Antibiotics+Catheter.Duration+Histology, data=turp, family=binomial())
summary(model) # AIC increased, so no need to consider duration over catheter provision
```

```
##
## Call:
## glm(formula = Bacteraemia ~ Prior.Antibiotics + Catheter.Duration +
##     Histology, family = binomial(), data = turp)
##
## Deviance Residuals:
##     Min       1Q   Median       3Q      Max
## -1.2002  -0.5669  -0.3233  -0.2400   2.6690
##
## Coefficients:
##                           Estimate Std. Error z value Pr(>|z|)
## (Intercept)                -3.5328     0.8874  -3.981 6.86e-05 ***
## Prior.Antibiotics           1.4235     0.6958   2.046   0.0408 *
## Catheter.DurationCISC       0.6077     1.4733   0.412   0.6800
## Catheter.DurationLT 1m      1.6713     0.9948   1.680   0.0929 .
## Catheter.DurationOver 1m    1.7859     0.9055   1.972   0.0486 *
## Histology                   1.5551     0.6826   2.278   0.0227 *
## ---
## Signif. codes:  0 '***' 0.001 '**' 0.01 '*' 0.05 '.' 0.1 ' ' 1
##
## (Dispersion parameter for binomial family taken to be 1)
##
```

```
##     Null deviance: 76.776  on 72  degrees of freedom
## Residual deviance: 59.111  on 67  degrees of freedom
## AIC: 71.111
##
## Number of Fisher Scoring iterations: 5
```
